# Supplementary material for: Expanding cholera serosurveillance to vaccinated populations
Source: medRxiv. 2025 Mar 11:2025.03.09.25323598. Preprint. [Version 1] doi: 10.1101/2025.03.09.25323598 (PMC11952589; doi:10.1101/2025.03.09.25323598)

medRxiv preprint doi: https://doi.org/10.1101/2025.03.09.25323598; this version posted March 11, 2025. The copyright holder for this preprint (which was not certified by peer review) is the author/funder, who has granted medRxiv a license to display the preprint in perpetuity.
It is made available under a CC-BY 4.0 International license .

Table S1. Timing of sample collection for each cohort

Exposure Type	Population	Days since exposure	Individuals	Samples (%)
Vaccination with Shanchol	Bangladeshi volunteer	0	43	43 (100)
Vaccination with Shanchol	Bangladeshi volunteer	3	43	40 (93)
Vaccination with Shanchol	Bangladeshi volunteer	14	43	40 (93)
Vaccination with Shanchol	Bangladeshi volunteer	17	43	40 (93)
Vaccination with Shanchol	Bangladeshi volunteer	28	43	37 (86)
Vaccination with Shanchol	Bangladeshi volunteer	42	43	36 (84)
Vaccination with Shanchol	Haitian volunteer	0	36	36 (100)
Vaccination with Shanchol	Haitian volunteer	7	36	36 (100)
Vaccination with Shanchol	Haitian volunteer	21	36	35 (97)
Vaccination with Shanchol	Haitian volunteer	44	36	22 (61)
Vaccination with Shanchol	Haitian volunteer	90	36	34 (94)
Vaccination with Shanchol	Haitian volunteer	180	36	18 (50)
Vaccination with Shanchol	Haitian volunteer	220	36	8 (22)
Vaccination with Shanchol	Haitian volunteer	360	36	23 (64)
Natural infection with *V. cholerae* O1	Bangladeshi case-patient	2	48	48 (100)

medRxiv preprint doi: https://doi.org/10.1101/2025.03.09.25323598; this version posted March 11, 2025. The copyright holder for this preprint (which was not certified by peer review) is the author/funder, who has granted medRxiv a license to display the preprint in perpetuity.
It is made available under a CC-BY 4.0 International license .

Exposure Type	Population	Days since exposure	Individuals	Samples (%)
Natural infection with *V. cholerae* O1	Bangladeshi case-patient	7	48	46 (96)
Natural infection with *V. cholerae* O1	Bangladeshi case-patient	30	48	46 (96)
Natural infection with *V. cholerae* O1	Bangladeshi case-patient	90	48	42 (88)
Natural infection with *V. cholerae* O1	Bangladeshi case-patient	180	48	40 (83)
Natural infection with *V. cholerae* O1	Bangladeshi case-patient	270	48	12 (25)
Natural infection with *V. cholerae* O1	Bangladeshi case-patient	360	48	14 (29)
Natural infection with *V. cholerae* O1	Bangladeshi case-patient	540	48	25 (52)
Natural infection with *V. cholerae* O1	Bangladeshi case-patient	720	48	1 (2)
Natural infection with *V. cholerae* O1	Bangladeshi case-patient	900	48	25 (52)
Natural infection with *V. cholerae* O1	Bangladeshi case-patient	1,080	48	1 (2)
Household member of case-patient infected with *V. cholerae* O1	Bangladeshi volunteer	2	3	3 (100)

medRxiv preprint doi: https://doi.org/10.1101/2025.03.09.25323598; this version posted March 11, 2025. The copyright holder for this preprint (which was not certified by peer review) is the author/funder, who has granted medRxiv a license to display the preprint in perpetuity.
It is made available under a CC-BY 4.0 International license .

Exposure Type	Population	Days since exposure	Individuals	Samples (%)
Household member of case-patient infected with *V. cholerae* O1	Bangladeshi volunteer	7	3	3 (100)
Household member of case-patient infected with *V. cholerae* O1	Bangladeshi volunteer	30	3	3 (100)

medRxiv preprint doi: https://doi.org/10.1101/2025.03.09.25323598; this version posted March 11, 2025. The copyright holder for this preprint (which was not certified by peer review) is the author/funder, who has granted medRxiv a license to display the preprint in perpetuity.
It is made available under a CC-BY 4.0 International license .

Figure S1. Baseline comparison of IgG markers

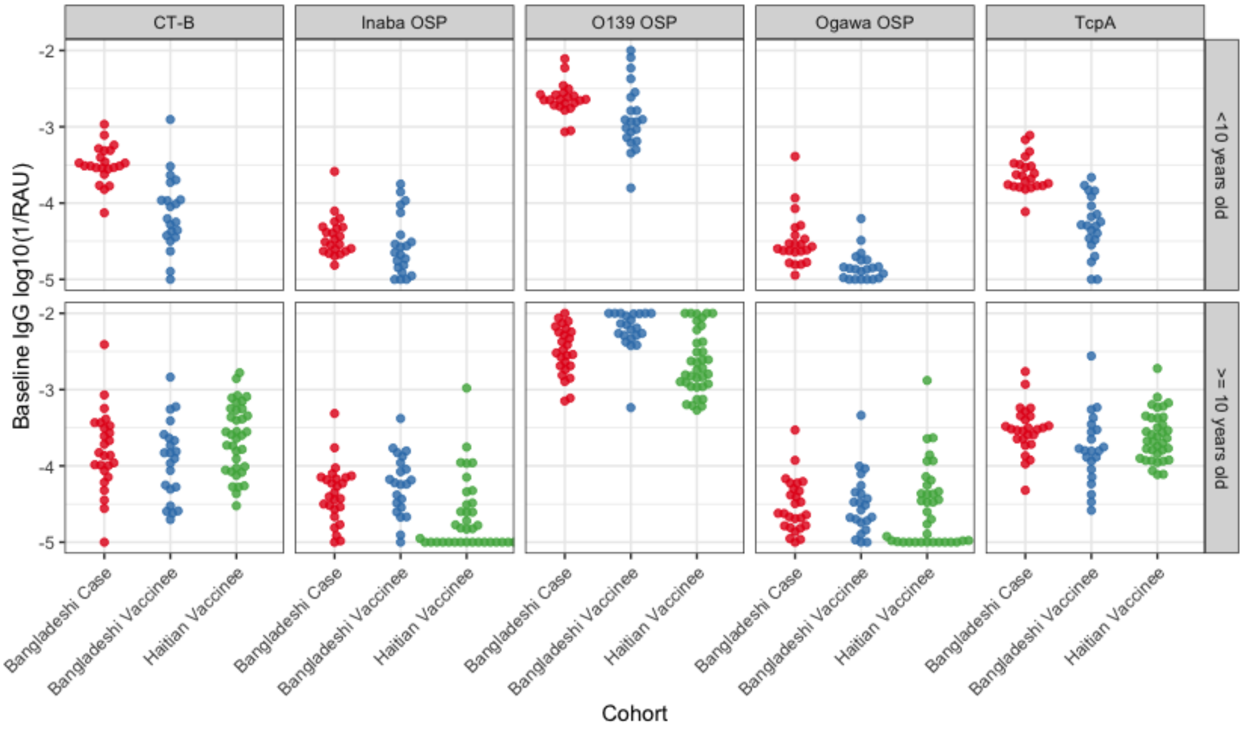

medRxiv preprint doi: https://doi.org/10.1101/2025.03.09.25323598; this version posted March 11, 2025. The copyright holder for this preprint
(which was not certified by peer review) is the author/funder, who has granted medRxiv a license to display the preprint in perpetuity.
It is made available under a CC-BY 4.0 International license .

Table S2. Geometric mean fold-rise in RAU by marker among cases and vaccines

Antigen	Isotype	Case	Vaccinee
CT-B	IgA	23.3	1.2
CT-B	IgG	23.9	1.5
CT-B	IgM	1.5	1.1
Inaba OSP	IgA	15.1	6.6
Inaba OSP	IgG	12.5	6.1
Inaba OSP	IgM	13.2	4.4
O139 OSP	IgA	2.1	2.0
O139 OSP	IgG	1.7	1.6
O139 OSP	IgM	1.5	1.4
Ogawa OSP	IgA	24.5	8.2
Ogawa OSP	IgG	33.4	9.7
Ogawa OSP	IgM	44.9	6.7
TcpA	IgA	3.4	1.3
TcpA	IgG	4.1	1.7
TcpA	IgM	1.8	1.2

medRxiv preprint doi: https://doi.org/10.1101/2025.03.09.25323598; this version posted March 11, 2025. The copyright holder for this preprint (which was not certified by peer review) is the author/funder, who has granted medRxiv a license to display the preprint in perpetuity.
It is made available under a CC-BY 4.0 International license .

Table S3. Proportion of individuals with a two fold-rise in RAU by marker among cases and vaccines

Antigen	Isotype	Case	Vaccinee
CT-B	IgA	100%	10%
CT-B	IgG	100%	23%
CT-B	IgM	25%	4%
Inaba OSP	IgA	81%	70%
Inaba OSP	IgG	85%	70%
Inaba OSP	IgM	92%	72%
O139 OSP	IgA	48%	42%
O139 OSP	IgG	31%	22%
O139 OSP	IgM	21%	18%
Ogawa OSP	IgA	85%	81%
Ogawa OSP	IgG	92%	68%
Ogawa OSP	IgM	92%	85%
TcpA	IgA	67%	18%
TcpA	IgG	75%	30%
TcpA	IgM	33%	8%

medRxiv preprint doi: https://doi.org/10.1101/2025.03.09.25323598; this version posted March 11, 2025. The copyright holder for this preprint (which was not certified by peer review) is the author/funder, who has granted medRxiv a license to display the preprint in perpetuity.
It is made available under a CC-BY 4.0 International license .

Figure S2: Multidimensional scaling analysis of all serological data collected

Each panel includes the two dimensions calculated from data collected within the specified time window. Black points represent the location of the centroids for the two new dimensions of cases (circle) and vaccinees (triangle).

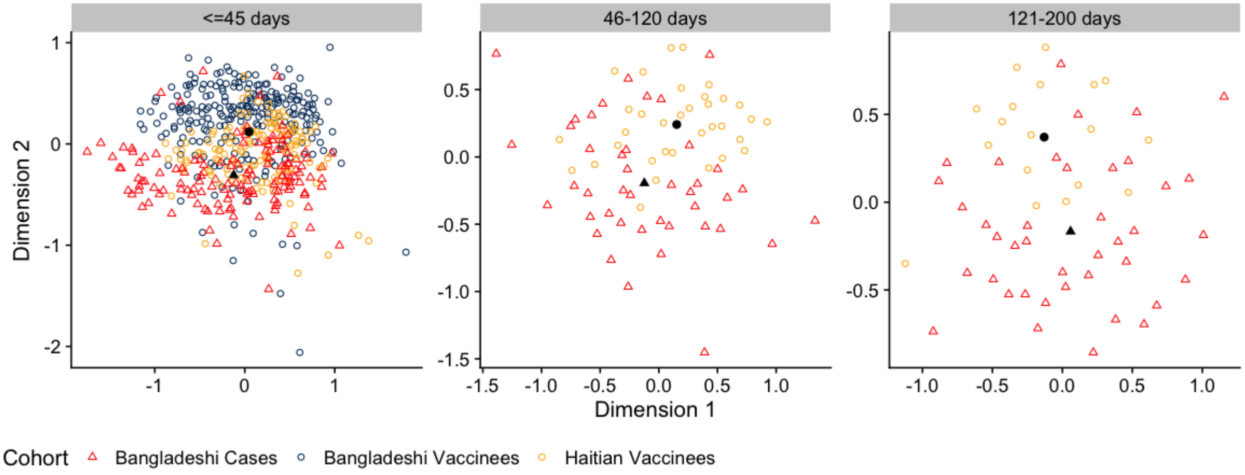

medRxiv preprint doi: https://doi.org/10.1101/2025.03.09.25323598; this version posted March 11, 2025. The copyright holder for this preprint (which was not certified by peer review) is the author/funder, who has granted medRxiv a license to display the preprint in perpetuity. It is made available under a CC-BY 4.0 International license .

Figure S3: Misclassification of vaccinees as seroincident by previous seroincidence models with additional markers using 45, 120, 200, and 300 day infection windows. The model used (i.e., the Case Only Model) to classify vaccinees as seroincident or not was a previously published random forest model trained on anti-CTB, anti-Ogawa OSP, anti-Inaba OSP, anti-TcpA antibodies (IgG, IgM, and IgA) as well as anti-O139 OSP IgG from Bangladeshi confirmed cases and uninfected household contacts. The proportion of Bangladeshi (dark blue) and Haitian vaccinees (gold) classified as seroincident are shown as dots. The overall proportion seroincident was modeled with a cubic spline (black line and grey ribbon) using data from both cohorts of vaccinees. Black dashed line indicates the nominal false positivity rate of 5%.

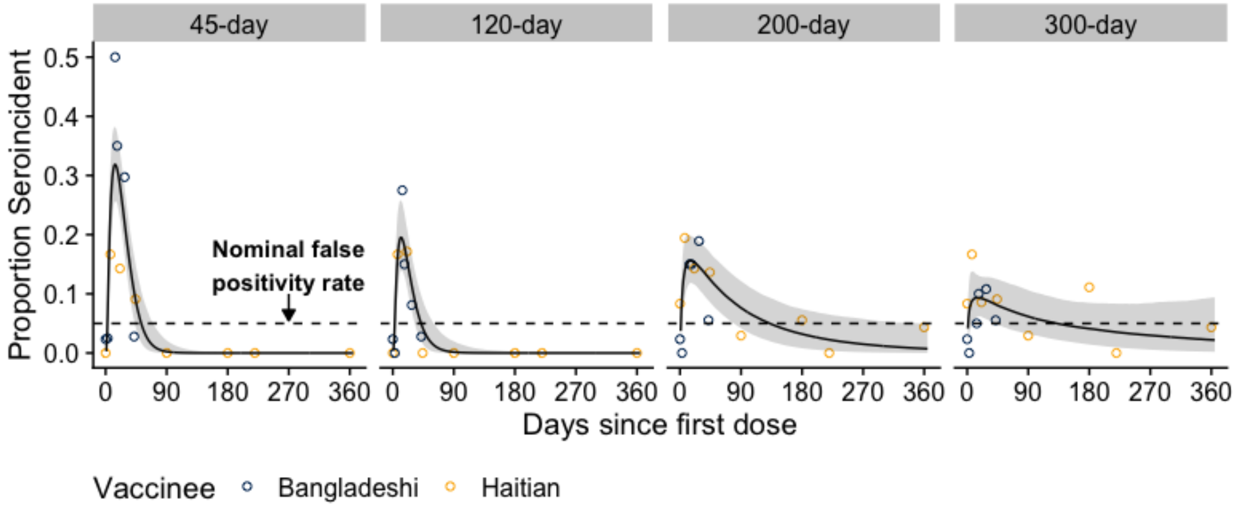

medRxiv preprint doi: https://doi.org/10.1101/2025.03.09.25323598; this version posted March 11, 2025. The copyright holder for this preprint (which was not certified by peer review) is the author/funder, who has granted medRxiv a license to display the preprint in perpetuity.
It is made available under a CC-BY 4.0 International license .

Figure S4: Comparison of performance of random forest models when all IgG, IgM, and IgA serological data from vaccinees are included in the training set. Individuals were considered recently infected or vaccinated if exposed in the last 200 days. (A & B) Grey dashed line indicates the expected/nominal false positivity rate of 5%. (A & C) Solid lines show the median value while shaded areas indicate the 95% credible interval. (D) Confusion matrix indicates the proportion of samples correctly classified from the new three-class model (*Mixed-Cohort Three Class Model*). Aside from the estimates for the false positivity rate among the vaccinated population for the *Case-only Model*, all other parameters were estimated through leave-one-individual-out cross-validation.

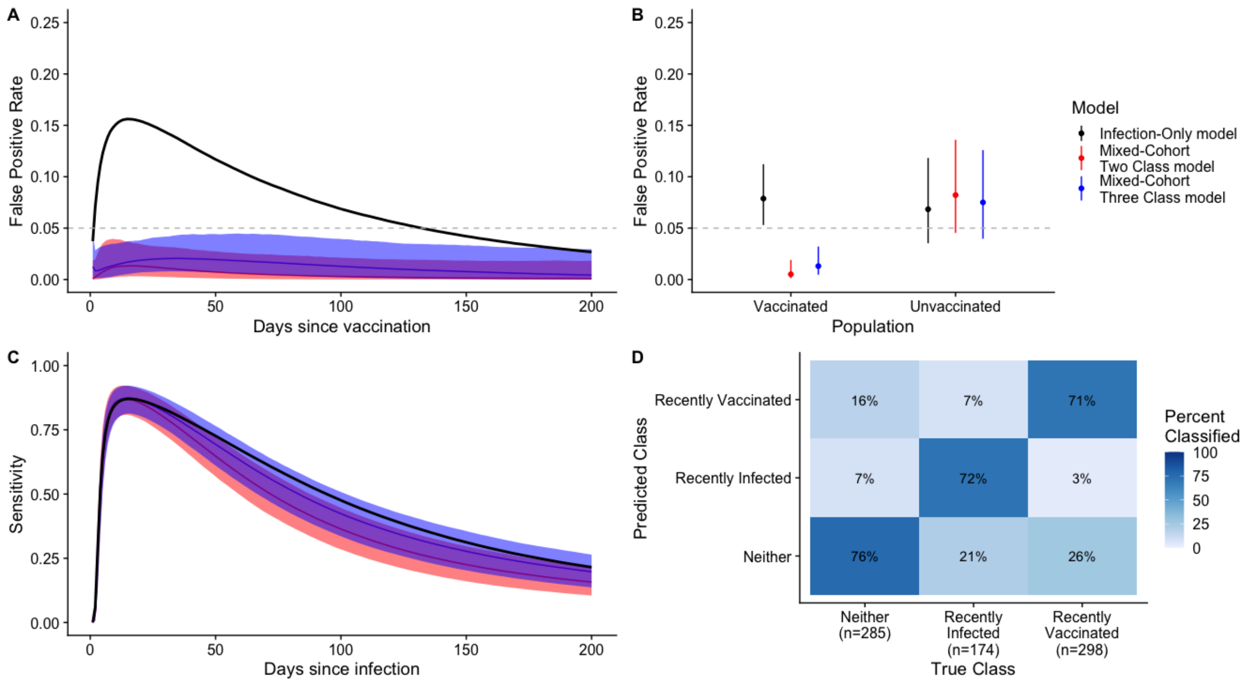

Supplement: 1 [file NIHPP2025.03.09.25323598V1-supplement-1.pdf]
